# Elongator function in tRNA wobble uridine modification is conserved between yeast and plants

**DOI:** 10.1111/j.1365-2958.2010.07163.x

**Published:** 2010-04-26

**Authors:** Constance Mehlgarten, Daniel Jablonowski, Uta Wrackmeyer, Susan Tschitschmann, David Sondermann, Gunilla Jäger, Zhizhong Gong, Anders S Byström, Raffael Schaffrath, Karin D Breunig

**Affiliations:** 1 Institut für Biologie, Genetik, Martin-Luther-Universität Halle-WittenbergWeinbergweg 10, D-06120 Halle (Saale), Germany; 2 Department of Molecular Biology, Umea University90187 Umea, Sweden; 3 State Key Laboratory of Plant Physiology and Biochemistry, College of Biological Sciences, China Agricultural UniversityBeijing 100094, China

## Abstract

Based on studies in yeast and mammalian cells the Elongator complex has been implicated in functions as diverse as histone acetylation, polarized protein trafficking and tRNA modification. Here we show that *Arabidopsis* mutants lacking the Elongator subunit AtELP3/ELO3 have a defect in tRNA wobble uridine modification. Moreover, we demonstrate that yeast *elp3* and *elp1* mutants expressing the respective *Arabidopsis* Elongator homologues *AtELP3/ELO3* and *AtELP1/ELO2* assemble integer Elongator complexes indicating a high degree of structural conservation. Surprisingly, *in vivo* complementation studies based on Elongator-dependent tRNA nonsense suppression and zymocin tRNase toxin assays indicated that while *AtELP1* rescued defects of a yeast *elp1* mutant, the most conserved Elongator gene *AtELP3*, failed to complement an *elp3* mutant. This lack of complementation is due to incompatibility with yeast *ELP1* as coexpression of both plant genes in an *elp1 elp3* yeast mutant restored Elongator's tRNA modification function *in vivo*. Similarly, *AtELP1*, not *ScELP1* also supported partial complementation by yeast–plant Elp3 hybrids suggesting that AtElp1 has less stringent sequence requirements for Elp3 than ScElp1. We conclude that yeast and plant Elongator share tRNA modification roles and propose that this function might be conserved in Elongator from all eukaryotic kingdoms of life.

## Introduction

Studies in fields as disparate as leaf development (Nelissen *et al*., 2003; 2005;) and drought resistance (Chen *et al*., 2006) in plants, neurodegeneration in humans (Anderson *et al*., 2001; Slaugenhaupt *et al*., 2001), cytotoxicity of a fungal toxin (Frohloff *et al*., 2001) and transcription elongation (Otero *et al*., 1999) have surprisingly converged on a conserved protein complex termed Elongator. The protein complex is composed of two subcomplexes with subunits Elp1, Elp2, and Elp3 as well as Elp4, Elp5 and Elp6 forming the hexameric holo-Elongator (Petrakis *et al*., 2004). Elp3 has a GNAT-type histone acetyltransferase (HAT) domain and has also been named Kat9 K-acetyltransferase to conform with recent acetyltransferase nomenclature (Allis *et al*., 2007). The Elongator complex was initially identified in yeast to interact with hyperphosphorylated RNA polymerase II and implicated in chromatin modification during transcription elongation (Otero *et al*., 1999; Wittschieben *et al*., 1999; Kristjuhan *et al*., 2002; Winkler *et al*., 2002; Gilbert *et al*., 2004; Kristjuhan and Svejstrup, 2004).

Surprisingly, it turned out that yeast Elongator mutants defective in any of the Elongator subunit genes (*ELP1* to *ELP6*) are lacking tRNA modifications at wobble uridines or thiouridines in position 34 of the anticodon (Huang *et al*., 2005). One of these tRNA modifications, 5-methoxycarbonyl-methyl-2-thiouridine (mcm^5^s^2^U), renders *Saccharomyces cerevisiae* sensitive to a toxin (zymocin) secreted by *Kluyveromyces lactis* (reviewed in Schaffrath and Breunig, 2000) and leads to the identification of *ELP/TOT* genes in a screen for zymocin-resistant *t*arget *o*f *t*oxin (*tot*) mutants (Frohloff *et al*., 2001). The γ-subunit (γ-toxin) of the zymocin is a tRNA endonuclease that cleaves a subset of tRNAs carrying the modified wobble uridine (mcm^5^s^2^U) (Huang *et al*., 2005; Lu *et al*., 2005; 2008; Jablonowski *et al*., 2006).

Genomic sequence analysis suggests that an Elongator complex exists in all eukaryotes. In plants, Elongator mutants have been isolated in a screen for mutants with aberrant leaf morphology. The *Arabidopsis thaliana elongata* (*elo*) class of leaf mutants consisting of four loci, *elo1* to *elo4*, is characterized by narrow and elongated shape of leaves and deficiencies in organ growth. *ELO1* identified the *Arabidopsis* homologue of yeast *ELP4*, *ELO2* is homologous to *ELP1*, and *ELO3* to *ELP3* (Nelissen *et al*., 2005). Moreover, the *DEFORMED ROOTS AND LEAVES* (*DRL1*) gene is allelic to *elo4* and shows sequence similarity to yeast *KTI12*, which encodes an Elongator partner protein (Fichtner *et al*., 2002a; Nelissen *et al*., 2003). The similar phenotypes of these mutants support the view that the gene products function in a complex and suggest that plant Elongator is similar to the one in yeast.

So far the molecular function of the complex is not well defined. Apparently, its acetyltransferase activity is essential but the substrate spectrum may be wider than initially anticipated (Svejstrup, 2007). Phenotypes associated with Elongator deficiency range from impaired zygotic paternal genome demethylation (Okada *et al*., 2010) to altered microtubule dynamics in human and *Caenorhabditis elegans* (Creppe *et al*., 2009; Solinger *et al*., 2010). Recently, mammalian Elongator was linked to acetylation of α-tubulin and shown to affect the migration and differentiation of cortical neurons (Creppe *et al*., 2009). In *C. elegans* Elongator deficiency was also associated with neurological and developmental defects and tRNA modification defects (Chen *et al*., 2009). *C. elegans* mutants still contain acetylated α-tubulin although the level may be reduced (Chen *et al*., 2009; Solinger *et al*., 2010). It has also been questioned whether histones are primary substrates for the acetyltransfer reaction because transcription-related phenotypes in yeast Elongator mutants could be suppressed by tRNA overexpression (Esberg *et al*., 2006).

Whether protein acetylation is the primary and only biochemical activity of Elongator in eukaryotes is unknown. The pleiotropic phenotypes of yeast Elongator mutants in processes as diverse as translation, exocytosis, filamentous growth and transcriptional silencing (Rahl *et al*., 2005; Johansson *et al*., 2008; Abdullah and Cullen, 2009; Li *et al*., 2009) might be explained by consequential effects of improper tRNA modification or by multiple acetylation substrates for Elongator, but there is also evidence for additional biochemical activities (Huang *et al*., 2005; Li *et al*., 2009; Lipardi and Paterson, 2009).

Here we have addressed the question whether the tRNA modification function is conserved between yeast and plants. We show that in an *A. thaliana elp3* mutant, tRNA wobble uridine modifications including mcm^5^s^2^U are compromised. By complementing yeast *elp* mutants with *A. thaliana ELO/AtELP* genes we demonstrate that the yeast subunits can assemble with plant polypeptides to form hybrid Elongator complexes indicating high structural similarity between yeast and plant Elongator. Strikingly, despite the fact that AtElp3/ELO3, the most conserved subunit, could structurally replace yeast Elp3 functional complementation with *AtELP3/ELO3* was not observed unless *ELP1* was simultaneously replaced by *AtELP1/ELO2*. Taken together the data strongly support the view that the tRNA modification function of Elongator is conserved between yeast and plants and most likely among all eukaryotes.

## Results

### Formation of a yeast Elongator complex containing AtELP3

**Fig. 1:**
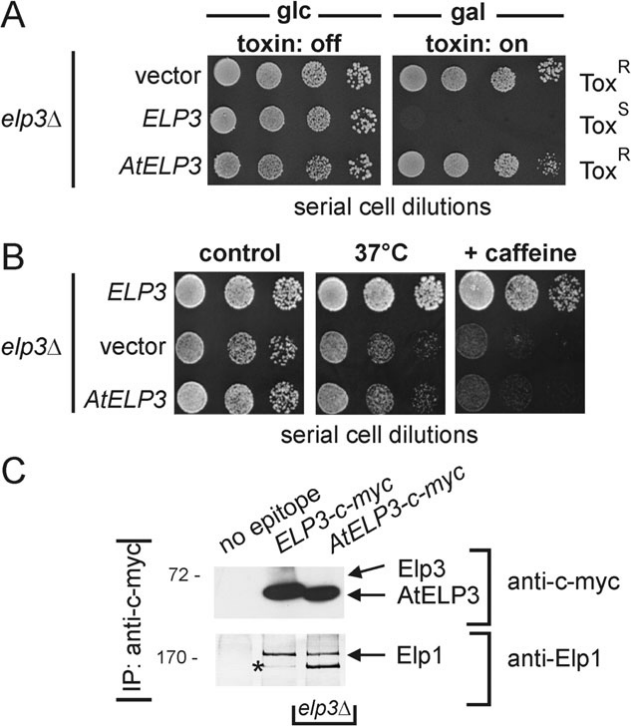
Failure of *AtELP3* to complement the *elp3*Δ mutant despite interaction with yeast Elp1 protein. A. To test for functional complementation the *elp3*Δ mutant (CMY135) was transformed with plasmids containing *ELP3* (pFF9), *AtELP3* (YEpA4) and vector control (YEplac195) and subsequently with the *GAL1* promoter driven γ-toxin expression plasmid pHMS14. Transformants were spotted in replica onto glucose-repressing (glc) or galactose-inducing (gal) media and grown for 4 days at 30°C. Growth on galactose indicates γ-toxin resistance (Tox^R^) and no growth corresponds to γ-toxin sensitivity (Tox^S^). B. To test for thermosensitivity and hypersensitivity to caffeine strains were serially diluted and replica spotted on YPD plates lacking (control) or containing 7.5 mM caffeine (right) and incubated for 4 days at 30°C and 37°C (middle). C. Anti-c-myc immunoprecipitates (IP) of strains containing chromosomally tagged *ELP3-c-myc* (FFY3t), *AtELP3-c-myc* on a plasmid in an *elp3*Δ background (CMY307 + yatELP3M) and wild-type parent without epitope (FY1679-8A) were analysed on Western blots probed with anti-c-myc or anti-Elp1 antibodies. Positions of the respective proteins are indicated by arrows. Protein extracts from cells without epitope-tag served as negative control. The asterisk denotes an N-terminally truncated Elp1 form (Fichtner *et al*., 2003). The relative abundance of this truncated form varies between experiments but is probably not dependent on which Elp3 peptide is present.

The *A. thaliana* genome contains one and only one homologue for each of the yeast *ELP* genes and evidence for a similar hexameric complex, composed of two subcomplexes, was recently obtained by tandem affinity purification (Nelissen *et al*., 2010). We tried to complement yeast *elp* mutant strains with the corresponding plant cDNAs fused to a yeast promoter. Sensitivity to γ-toxin, the active component of the *K. lactis* killer toxin zymocin provided a sensitive assay for Elongator function. If the heterologous protein integrated into the yeast Elongator complex and functioned in restoring tRNA modification, we expected reversion of the toxin resistance phenotype of the Elongator mutant. Because the AtELP3 subunit is most similar to its yeast homologue, we first tried to complement the *elp3* mutant. A c-myc-tagged version of the AtELP3 protein could be produced at levels comparable to those of yeast Elp3-c-myc (see below), but the toxin resistance of the yeast *elp3*Δ mutant was unaffected by the *AtELP3-c-myc* gene (not shown) or an untagged *AtELP3* allele (Fig. 1A). Likewise, thermosensitivity and hypersensitivity to caffeine, additional phenotypes of Elongator mutants, were not altered by the plant gene (Fig. 1B). Reintroduction of the yeast *ELP3* gene into the *elp3*Δ reporter, however, fully complemented all three phenotypes (Fig. 1A and B).

The failure of plant AtELP3 to substitute for yeast Elp3 function was not due to instability of the protein as AtELP3-c-myc was precipitated from total yeast protein extracts at levels comparable to Elp3-c-myc (Fig. 1C, top panel). c-myc-tagged Elp3 and AtElp3 both could co-precipitate the largest Elongator subunit Elp1 indicating interaction between AtELP3 and Elp1 (Fig. 1C, bottom panel).

**Fig. 2:**
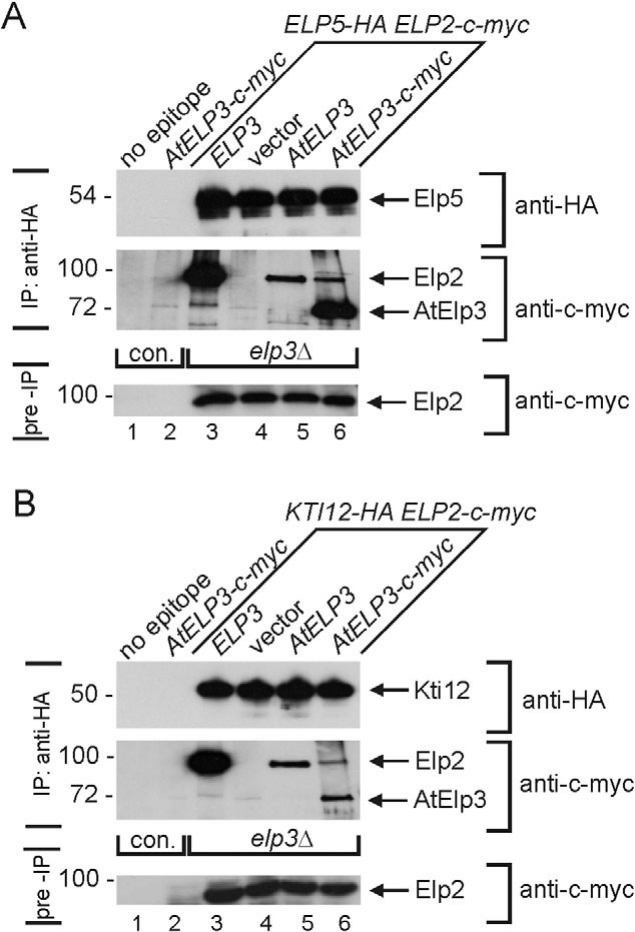
Reconstitution of Elongator subunit interactions by AtELP3 demonstrating AtELP3 integration in the yeast Elongator complex. A. Protein extracts from cells expressing HA-tagged Elp5 together with c-myc-tagged Elp2 in an *elp3*Δ background (strains CMY304 and CMY301, respectively) and transformed with plasmids containing *ELP3* (pFF9), *AtELP3* (YEpA4), *AtELP3-c-myc* (yatELP3M) or vector control (YEplac195) were immunoprecipitated using anti-HA antibody and analysed by Western blotting. B. Same as (A) but with strain CMY301, which expresses HA-tagged Kti12 instead of Elp5-HA. The anti-HA antibody was used to detect Elp5-HA or Kti12-HA and anti-c-myc antibodies recognized Elp2 and AtELP3. Protein extracts from cells without epitope tag and cells expressing only AtELP3-c-myc served as negative controls. The pre-IPs served as loading control (bottom panels).

To analyse whether AtELP3-Elp1 interaction occurred in the context of the Elongator complex we made use of the fact that interaction between the subunits Elp5 and Elp2 depends on the structural integrity of the complex and the presence of Elp3 (Frohloff *et al*., 2003; Petrakis *et al*., 2004). We constructed *elp3*Δ reporter strains expressing a c-myc-tagged version of Elp2 and an HA-tagged version of Elp5. As expected, co-immunoprecipitation of Elp2-c-myc with Elp5-HA was not observed when Elp3 was lacking (Fig. 2A, lane 4) but was found when the *elp3*Δ mutant was complemented with the yeast *ELP3* gene on a plasmid (Fig. 2A, lane 3). When *AtELP3* or *AtELP3-c-myc* alleles were introduced instead of *ELP3*, pull-down of Elp2-c-myc by Elp5-HA was less efficient (Fig. 2A, lanes 5 and 6), but significantly higher than in the empty vector control (Fig. 2A, lane 4). Thus, plant AtELP3 promotes Elp5-Elp2 interaction in a yeast *elp3*Δ mutant background.

Using the same approach we also tested whether the chimeric complex was able to interact with Kti12, a protein that associates with the Elongator complex (Fichtner *et al*., 2002a; Petrakis *et al*., 2005). As with Elp5-HA, Kti12-HA was able to pull-down Elp2-c-myc in the presence but not in the absence of yeast Elp3 (Fig. 2B, lanes 3 and 4) and again AtELP3 could substitute for yeast Elp3 in this interaction assay (Fig. 2B, lanes 5 and 6). The latter finding is particularly intriguing because Elp2–Kti12 interaction relies on the association between the two Elongator subcomplexes Elp1–Elp2–Elp3 and Elp4–Elp5–Elp6 (Fichtner *et al*., 2002b; Frohloff *et al*., 2003). We conclude that AtELP3 can structurally replace Elp3 in the yeast Elongator complex but apparently the complex is not functional.

### Plant AtELP1 restores Elongator subunit interactions in an *elp1*Δ yeast mutant

**Fig. 3:**
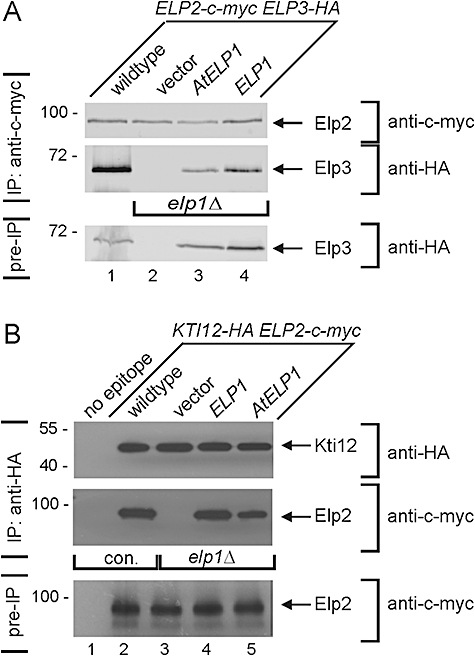
Restoration of Elp3 stability and Elongator subunit interactions in an *elp1*Δ strain expressing AtElp1. A. AtElp1 is able to replace yeast Elp1 and to support Elp2–Elp3 interaction and Elp3 protein stability. Protein extracts from cells expressing chromosomally c-myc-tagged Elp2 together with HA-tagged Elp3 in an *elp1*Δ background (FFY2/3-dt-1d) and transformed with plasmids containing *ELP1* (pFF13), *AtELP1* (pDJ98) or vector control (YEplac181) were immunoprecipitated using anti-c-myc antibodies. Western blots of the precipitates were probed with anti-c-myc or anti-HA antibodies to detect Elp2 and Elp3 respectively (arrows). Instability of Elp3 in the *elp1*Δ strain is revealed in pre-IPs (bottom panel, lane 3). B. AtElp1 is able to restore Kti12-Elp2 interaction. Protein extracts from cells expressing c-myc-tagged Elp2 together with HA-tagged Kti12 in an *elp1*Δ background (CMY300), and transformed with plasmids containing *ELP1* (pFF13), *AtELP1* (pDJ98) or vector control (YEplac181) were immunoprecipitated and analysed as in (A).

Incorporation of AtElp3 into yeast Elongator requires interaction with much less conserved components. Elp1 and AtELP1 display only 19% amino acid identity compared with 67% between Elp3 and AtELP3. To analyse whether AtELP1 could also structurally replace yeast Elp1 in the complex, an *elp1*Δ strain that contained the epitope-tagged Elongator subunits Elp3-HA and Elp2-c-myc was transformed with an *AtELP1* cDNA clone. Consistent with previous reports (Frohloff *et al*., 2003; Petrakis *et al*., 2004), we found that the Elp1 subunit is not only required for Elp3-Elp2 interaction (Fig. 3A, lane 2) but also for stability of yeast Elp3. Hence, an HA-tagged version of Elp3 was not detectable in total protein extracts of the *elp1*Δ mutant (Fig. 3A, bottom panel, lane 2). Reintroduction of the yeast *ELP1* gene restored Elp3-HA stability and interaction between Elp2 and Elp3 (Fig. 3A, lane 4). Remarkably, the same held true when *AtELP1* was introduced (Fig. 3A, lane 3). Expression of the plant gene from the inducible *GAL1* promoter in the *elp1*Δ background allowed Elp2-c-myc to precipitate Elp3-HA. *AtELP1* expression also restored the interaction between Elp2 and Kti12 (Fig. 3B).

Again, as in the *AtELP3*-expressing *elp3* mutant, the efficiency of subunit interactions was somewhat reduced compared with that of the *ELP1* transformants. Nonetheless, our data show that AtELP1 like AtELP3 are assembled into complexes where they are able to structurally replace the respective yeast Elongator subunits.

### Together, plant AtELP1 and AtELP3 support tRNA modification in yeast

**Fig. 4:**
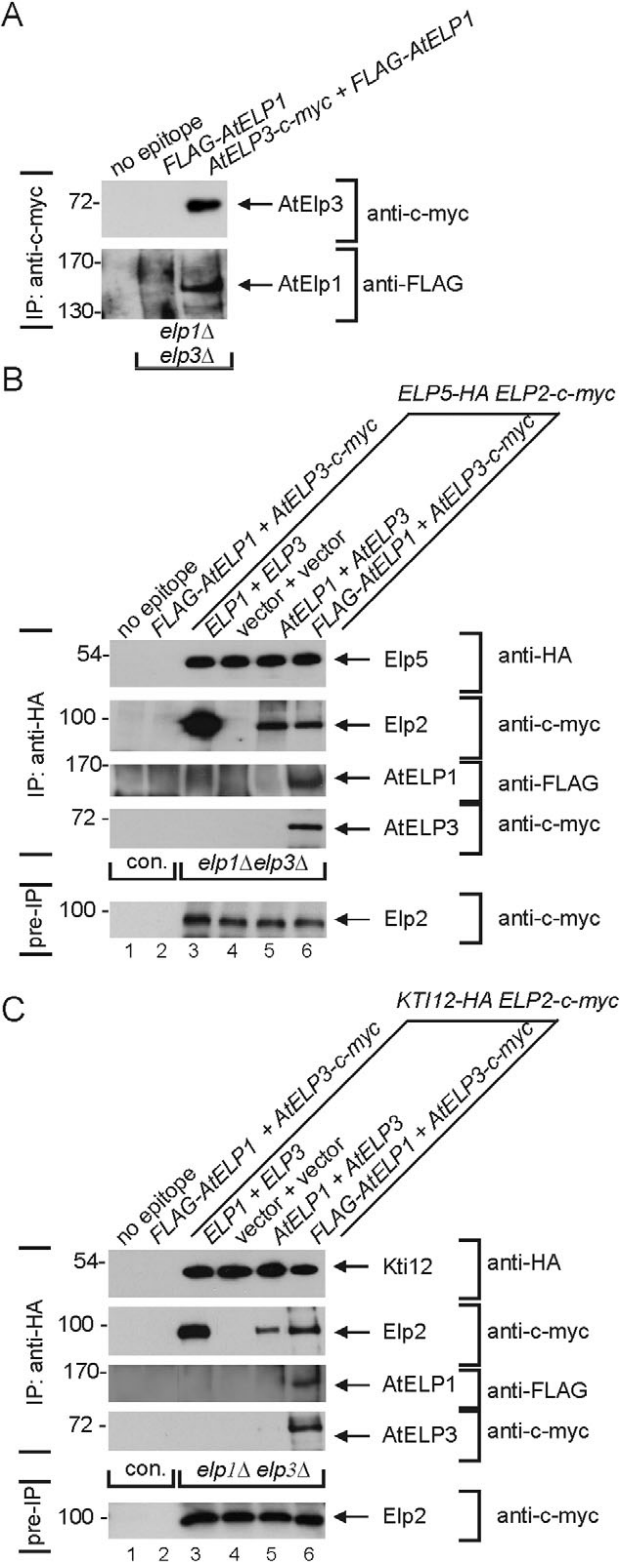
Formation of a chimeric Elongator complexes composed of yeast and plant subunits. A. AtELP1 and AtELP3 interact with each other in yeast. Anti-c-myc immunoprecipitates of *elp1*Δ *elp3*Δ strains (CMY134) containing *FLAG-AtELP1* (pJET13) and *AtELP3-c-myc* were probed with anti-c-myc antibodies to detect AtELP3-c-myc and with anti-FLAG-antibodies to detect FLAG-AtELP1. Immunoprecipitates with anti-c-myc antibodies from *elp1*Δ*elp3*Δ cells containing p*FLAG-AtELP1* (lane 2) or no epitope-tag (lane1) served as negative controls. B. AtELP1 and AtELP3 restore Elp2–Elp5 interaction in the *elp1*Δ *elp3*Δ mutant. Protein extracts from cells expressing HA-tagged Elp5 together with c-myc-tagged Elp2 in an *elp1*Δ *elp3*Δ double mutant background (CMY305) and transformed with plasmids containing *ELP1* (pFF13), *ELP3* (pFF9), *AtELP1* (pDJ98), *AtELP3* (YEpA4), *FLAG-AtELP1* (pJET13), *AtELP3-c-myc* (p*ELO3-myc*) and vector controls (YEplac181, YEplac195) were immunoprecipitated using anti-HA antibodies. The antibodies used to detect the indicated proteins by Western blotting are marked on the right. Protein extracts from cells without epitope-tag and cells expressing only *AtELP3-c-myc* served as negative controls. Pre-IPs served as loading control (bottom panel). C. AtELP1 and AtELP3 restore Elp2–Kti12 interaction. Protein extracts from strain CMY302 expressing HA-tagged Kti12 together with c-myc-tagged Elp2 in an *elp1*Δ *elp3*Δ background and transformed (see B) were analysed as described in (B).

**Fig. 5:**
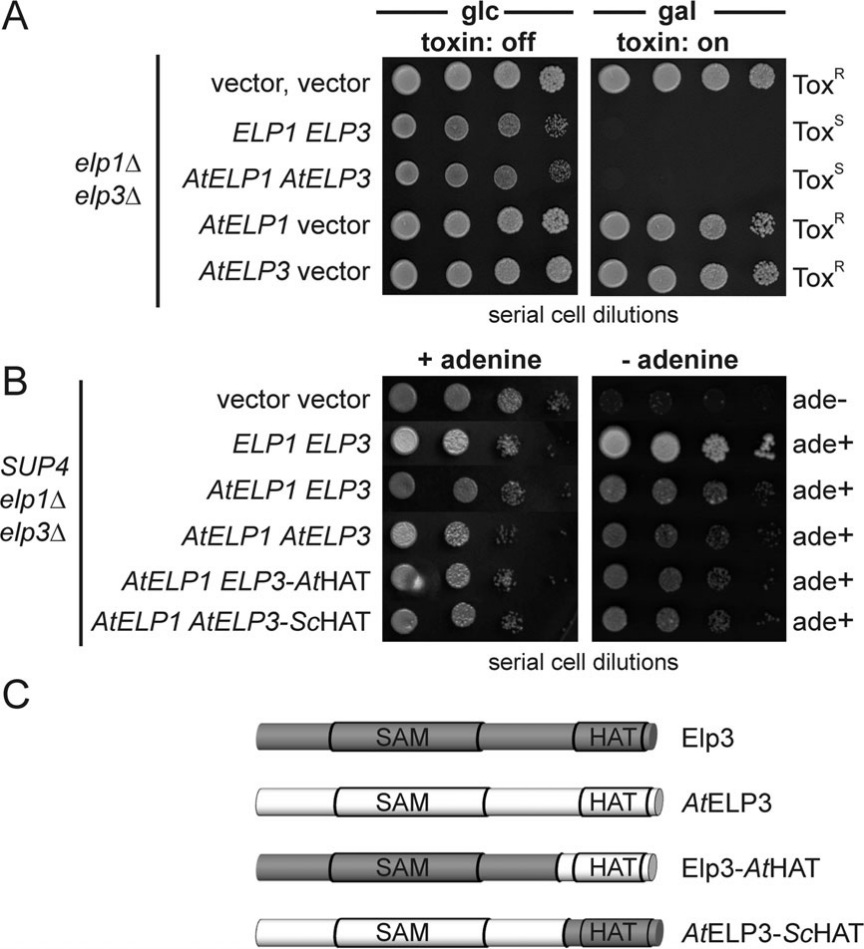
Restoration of the tRNA modification function of Elongator by complementation of a yeast *elp1*Δ*elp3*Δ double mutant with *AtELP1* and *AtELP3*. A. Restoration of toxin sensitivity. The *elp1*Δ *elp3*Δ (CMY134) strain was transformed with plasmids containing *ELP1* (pFF13), *ELP3* (pFF9), *AtELP1* (pCM26), *AtELP3* (YEpA4) and vector controls (YEplac181 and YEplac195) in the indicated combinations. The γ-toxin was expressed from pHMS14 (Frohloff *et al*., 2001) on galactose medium. Growth on galactose after 4 days at 30°C indicates γ-toxin resistance (Tox^R^) and no growth equals γ-toxin sensitivity (Tox^S^). B. Restoration of *SUP4* suppressor tRNA function. *SUP4 elp1*Δ *elp3*Δ (CMY160) cells were transformed with plasmids containing wild-type *ELP1* and *ELP3* (pFF13 and p424TDH-ScElp3myc), the respective empty vectors (YEplac181 and p424TDH), the plant homologues *AtELP1* and *AtELP3* (pCM26 and p424TDH-AtElp3myc) or *AtELP1* and the swapped HAT domain variants pElp3-*At*HAT and pAtElp3-*Sc*HAT. To check for suppression of the *ade2-1* (UAA) ochre mutation (Huang *et al*., 2005) serial dilutions of the transformants were spotted on SC and SC-Ade plates and cultivated for 4 days at 30°C. *SUP4* suppressor tRNA function results in growth on SC-Ade plates (Ade^+^ phenotype). C. Schematic representation of the Elp3 domain structure and domain swapped hybrids generated between AtELP3 and ScElp3 used as in (B).

Because Elp3 requires Elp1 for stability, its function may depend on specific contacts between these two proteins explaining the failure of *AtELP3* to functionally complement the *elp3*Δ mutant in combination with yeast Elp1. To address this possibility we tested whether AtELP3 might function in yeast when supplied with AtELP1. *FLAG-AtELP1* was expressed together with the *AtELP3-c-myc* gene in a yeast *elp1*Δ*elp3*Δ double mutant. FLAG-AtELP1 could be detected in the anti-c-myc precipitates together with AtELP3-c-myc (Fig. 4A). Like in the single mutants, interaction between the epitope-tagged subunits Elp5-HA and Elp2-c-myc (Fig. 4B) as well as Kti12-HA and Elp2-c-myc (Fig. 4C) could be restored in the double mutant by the simultaneous expression of untagged (lanes 5) or tagged (lanes 6) versions of AtELP1 and AtELP3. Elp2-c-myc (lanes 3, 5 and 6) and AtElp3-c-myc (lane 6) could be detected in the anti-HA precipitates of strains containing yeast (lane 3) or *A. thaliana* Elp1 and Elp3. We conclude that a chimeric yeast–plant Elongator complex can form under these conditions. Intriguingly, AtELP1 and AtELP3 together were able to restore γ-toxin sensitivity in the yeast *elp1 elp3* double mutant indicating that the chimeric Elongator complex was functional (Fig. 5A). Because toxin sensitivity requires the Elongator-dependent tRNA wobble uridine modification mcm^5^s^2^U (Lu *et al*., 2005), the *A. thaliana* subunits can reasonably be assumed to carry out a function in tRNA modification that is equivalent to that of the yeast homologues.

To support this conclusion, we used a second assay monitoring Elongator-dependent tRNA modification that is based on the *SUP4* suppressor tRNA gene (Huang *et al*., 2005). The *SUP4* allele codes for a UAA ochre suppressor tRNA^Tyr^ with a G34-to-U34 exchange in the anticodon. Suppression of ochre nonsense codons by *SUP4* requires Elongator-dependent wobble uridine modification (Huang *et al*., 2005). In an *elp1*Δ*elp3*Δ double deletion strain (CMY160) carrying the ochre mutation *ade2-1* the *SUP4* tRNA suppressor was inactive resulting in an Ade^-^ phenotype (Fig. 5B, vector control). Transformation with two plasmids encoding either yeast Elp1 and Elp3 or AtElp1 and AtElp3 restored *SUP4* tRNA suppression, allowing for *ade2-1* readthrough and growth on adenine-lacking medium. Although growth in the latter case was weak we conclude that the AtElp1-AtElp3 containing chimeric Elongator can functionally replace yeast Elongator and promote ochre suppression by *SUP4*. Yeast Elp1 with AtElp3 was inactive in the adenine growth assay (data not shown) as in the γ-toxin assay (Fig. 5A).

Elp3 contains two protein motifs that are found in other functional contexts, the C-terminal GNAT-like HAT domain related to Gcn5 histone acteyltransferase (Wittschieben *et al*., 1999) and a central domain found in radical S-adenosyl-L-methionine enzymes (radical SAM domain) (Chinenov, 2002; Paraskevopoulou *et al*., 2006). To test which part of AtElp3 is not compatible with ScElp1 we constructed two hybrid variants with the HAT domain of yeast Elp3 replaced by that of AtELP3 and *vice versa* (Fig. 5C)*.* Like AtELP3, both chimeric Elp3 variants were unable to complement the *elp1 elp3* double mutant in the presence of yeast Elp1 (data not shown). However, in combination with AtELP1, AtELP3 and both hybrid proteins allowed for weak growth on adenine-deficient medium indicative of *SUP4*-dependent ochre suppression of the *ade2-1* mutation (Fig. 5B). Apparently, AtELP1 has a less stringent requirement with respect to Elp3 sequence than ScElp1. The weak Ade^+^ phenotype in combination with AtELP1 but not with yeast ELP3 was highly reproducible; however, we could not confirm reconstitution of tRNA modification by the hybrid Elp3 variants using the γ-toxin assay (data not shown). Probably partial complementation results in hypomodification of tRNAs and gives zymocin resistance

### tRNA modification is affected in Elongator-deficient plant cells

**Fig. 6:**
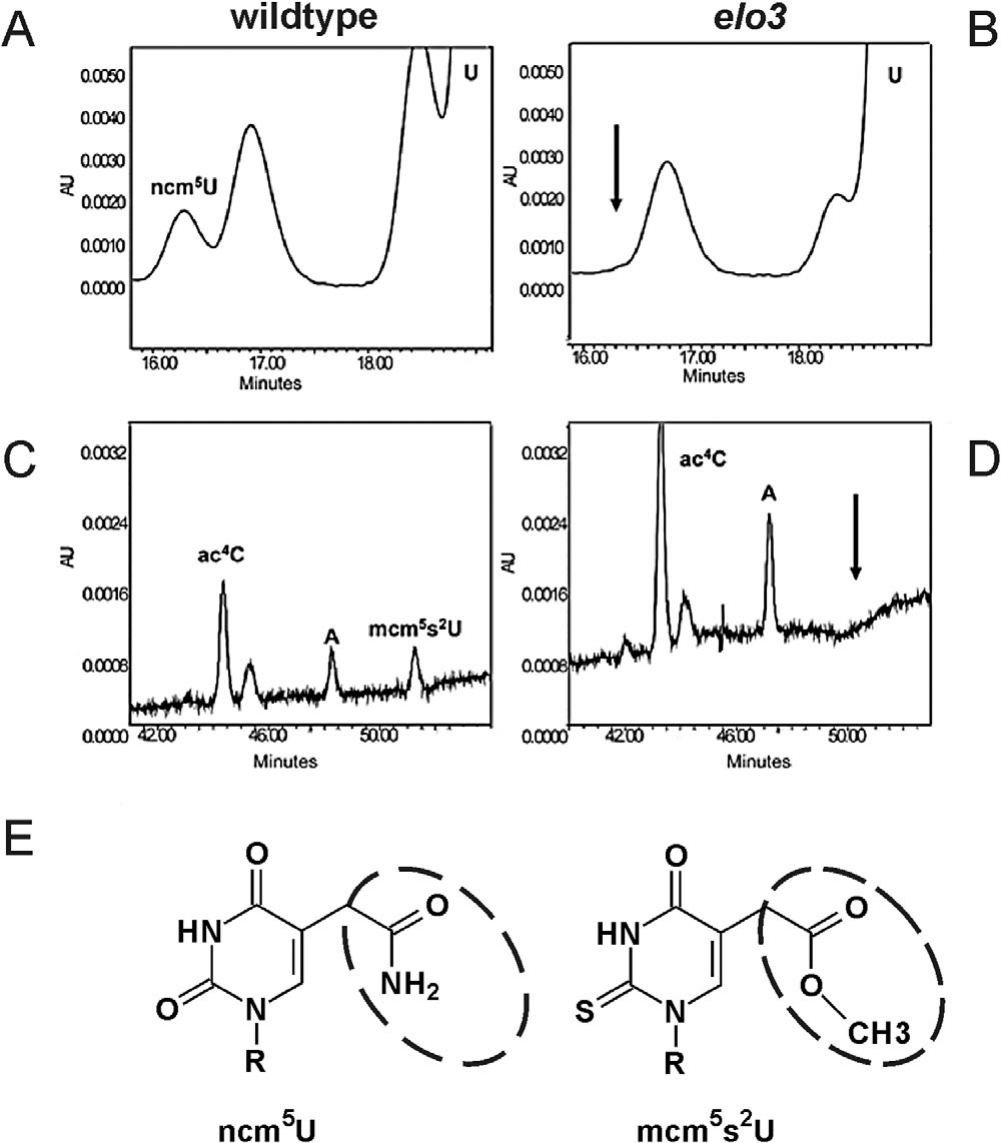
AtELP3/ELO3 is required for formation of mcm^5^s^2^U and ncm^5^U modified nucleosides in tRNA. A.–D. Total tRNA isolated from wild-type and *elo3* plants was analysed by HPLC (see *Experimental procedures*). Wild-type profiles are shown on the left, *elo3* profiles in the right panels. A. and B. The parts of the chromatograms between retention times 15.5 and 19.6 min are displayed. The arrow in (B) indicates the expected retention time of ncm^5^U. Chromatograms were monitored at 254 nm. C. and D. The parts of the chromatograms between retention times 40.0 and 52.5 min are displayed. The arrow in (D) indicates the expected retention time of mcm^5^s^2^U. Chromatograms were monitored at 314 nm. E. Uridine modifications found at the wobble position of eukaryotic tRNAs. Elongator-dependent side groups are circled (Huang *et al*., 2005).

Restoration of ochre suppression and γ-toxin sensitivity of the yeast *elp1*Δ*elp3*Δ double mutant by *AtELP1* and *AtELP3* suggested that plant Elongator may also affect tRNA modification in plant cells. For all but one tRNA modification genes of yeast (*TRM* genes) homologues can be found in plant genomes suggesting that plant tRNAs are modified in a similar way. To test whether Elongator deficiency in *A. thaliana* had any impact on tRNA modification RNA preparations enriched for small stable RNA were compared between wild-type and homozygous *elo*3 mutants. RNA was isolated from leaves of several independent plants, degraded to nucleosides and analysed by HPLC. The elution profiles were very similar for both samples except for the parts shown in Fig. 6, where 5-carbamoyl-methyluridine (ncm^5^U) (Fig. 6A and B) and mcm^5^s^2^U (Fig. 6C and D) eluted. Both modified uridine nucleosides (compare Fig. 6E) were lacking in the sample from the *elo3* mutant. These data clearly show that the *elo3* mutation prevented the ncm^5^ and mcm^5^s^2^-uridine modifications from being generated supporting the view that plant Elongator is involved in tRNA modification in a way similar to its yeast homologue. This indicated that the role of Elongator in tRNA modification might be conserved among lower and higher eukaryotes.

## Discussion

Biochemical characterizations of yeast and human Elongator and genomic sequences have indicated that the subunit composition of this complex is conserved among eukaryotes (reviewed in (Svejstrup, 2007). The data presented in this paper reinforce this view and provide evidence for a remarkable structural conservation between plant and yeast Elongator complexes. We further demonstrate that an *A. thaliana* Elongator mutant is lacking the very same tRNA modifications previously shown in yeast to be Elongator-dependent (Huang *et al*., 2005). Together with similar deficiencies reported for *C. elegans* Elongator mutants (Chen *et al*., 2009), these data strongly support the view that tRNA modification might be a function of Elongator that is conserved in animals, fungi and plants.

A crucial question is how Elongator affects tRNA modification. The acetyltransferase activity of the Elp3 subunit has been shown to be required (Huang *et al*., 2005). One possibility is that biosynthesis or activity of the enzymes generating the ncm^5^ or mcm^5^ side chains on the tRNA wobble uridines depend on acetylation. Alternatively, Elongator may directly participate in the tRNA modification reaction. The Elongator subunits Elp1 and Elp3 could be specifically cross-linked to tRNA^Glu^ but not tRNA^Met^ (Huang *et al*., 2005) supporting the latter model. A radical mechanism based on the putative radical SAM domain flanking the HAT motif in Elp3 (Chinenov, 2002; Paraskevopoulou *et al*., 2006) might be involved.

We have used yeast as a tool to analyse whether AtELP3 was able to function in the context of a heterologous Elongator complex. Two sensitive assays that allow to monitor the Elongator-dependent tRNA modification were used. The first one detects mcm^5^s^2^U-tRNA^Glu^ and, to a lesser extent, mcm^5^s^2^U-tRNA^Lys^ and mcm^5^s^2^U-tRNA^Gln^. These tRNAs are the primary targets of the tRNA endonuclease activity associated with the *K. lactis* zymocin. Defects in formation of the modified uridine and typical of Elongator mutants cause zymocin resistance (Lu *et al*., 2005; 2008; Jablonowski *et al*., 2006; Studte *et al*., 2008). Restoration of zymocin sensitivity in resistant mutants indicates efficient formation of the modified tRNAs. The second assay makes use of the *SUP4* ochre suppressor tRNA, which also depends on the mcm^5^ modification function of Elongator (Huang *et al*., 2005). Both assays are sensitive to a point mutation in the HAT domain of yeast Elp3 (Frohloff *et al*., 2001; Huang *et al*., 2005). Under conditions of partial complementation the two assays are expected to differ in sensitivity. Hypomodified tRNAs would confer (some degree of) resistance in the zymocin assay (i.e. the mutant phenotype) by escaping endonucleolytic cleavage but might support some growth on selective plates (the wild-type phenotype) in the nonsense suppression assay.

We have shown here that a double mutant lacking Elp3 and Elp1 can be complemented by the *Arabidopsis* homologues *AtELP1* and *AtELP3/ELO3*, which together restore zymocin sensitivity and *SUP4*-mediated nonsense suppression. Thus, these two subunits cooperate to functionally replace yeast Elp1 and Elp3 indicating that they have a similar role in the plant Elongator complex.

Surprisingly, despite 67% sequence identity between ScElp3 and AtELP3, the latter was unable to complement the yeast *elp3* mutation. Only in combination with AtELP1 could complementation of the Elongator mutant phenotypes by the *AtELP3* genes be achieved. AtELP3-c-myc alone was incorporated into the yeast complex as shown by its ability to restore association between the large and small subcomplexes and between Elongator and its partner protein Kti12. Both types of interactions require Elp3 and the structural integrity of the complex (Frohloff *et al*., 2001; Fichtner *et al*., 2002a; Fichtner *et al*., 2003; Petrakis *et al*., 2005). We conclude that, although an Elongator complex in which AtELP3 replaces Elp3 appears structurally intact, the AtELP3 containing chimeric complex is unable to provide the activity required for tRNA modification.

The lack of functional complementation by AtELP3 is probably not due to the reduced efficiency of Elp2–Elp5 or Elp2–Kti12 interactions as the same reduction was observed in the AtELP3 and AtELP1 containing chimeric complex, which is functional. AtELP3 appears stable in yeast in the presence of ScElp1 excluding the possibility that lack of function is due to protein degradation, which is observed for ScElp3 in the absence of ScElp1 (Fig. 3).

To determine whether sequence divergence in the HAT domain was responsible for the lack of complementation, domain swapped variants with *Sc*HAT replaced by *At*HAT and *vice versa* were tested*.* Like AtELP3, both variants were inactive in the presence of ScELP1. This failure of the *At*HAT to replace the corresponding yeast domain contrasts with the ability of the HsELP3-HAT to do so (Li *et al*., 2005). However surprisingly, in the *SUP4* tRNA suppression assay (but not in the zymocin assay), both hybrid *ELP3* genes were able to partially complement the yeast *elp3* mutation in combination with *AtELP1.* This indicates weak U34-tRNA modification mediated by AtELP1 in combination with multiple structural variants of Elp3 including ScElp3 (Chen *et al*., 2006). AtELP1 appears more tolerant towards sequence variations in the Elp3 protein. The crucial role of ScElp1 phosphorylation/dephosphorylation (Mehlgarten *et al*., 2009) might be important in this context. Alternatively but less likely, AtELP1 may contribute to complementation in yeast via an additional function not found in ScElp1, like the RNA-dependent RNA polymerase activity recently reported for *Drosophila* Elp1 (Lipardi and Paterson, 2009).

A current challenge is to understand the biological function(s) of the Elongator complexes in the various organisms and their possible divergence during evolution. If, as proposed here and supported by studies in *C. elegans* (Chen *et al*., 2009), tRNA modification defects are a general consequence of Elongator deficiency in eukaryotes it will be essential to determine whether the mutant phenotypes are causally related to this defect. Studies in yeast support such a view. Pleiotropic phenotypes of yeast Elongator mutants including hypoacetylation of histones, delayed adaptation of transcription to changing environmental conditions as well as delocalization of the secretory protein Sec2 could all be suppressed by overexpression of two tRNAs, tRNA^Lys^ and tRNA^Gln^, both of which in wild-type cells undergo Elongator-dependent anticodon modifications (Esberg *et al*., 2006). Based on these data it has been proposed that the effects of Elongator mutants on transcription and secretion are indirect consequences of influences on translation caused by inappropriate tRNA modification. Apparently, U34 modifications in the anticodon increase the decoding efficiency of A- and G-ending codons (Yokoyama *et al*., 1985; Lim, 1994; Durant *et al*., 2005; Esberg *et al*., 2006; Begley *et al*., 2007; Johansson *et al*., 2008). Thus, the translation of individual proteins might be differentially affected depending on their amino acid composition and codon usage. Moreover, yeast *elp* mutants are synthetic lethal with mutations affecting thiolation of U34 (Esberg *et al*., 2006; Johansson *et al*., 2008) emphasizing the importance of the wobble uridine modifications.

A causal relationship between tRNA hypomodification and Elongator phenotypes does not exclude that Elongator has additional activities or multiple targets for the acetyltransferase reaction. In plants, yeast and human cells the complex is also found in the nucleus and its depletion affects transcription (Winkler *et al*., 2002; Close *et al*., 2006; Svejstrup, 2007; Nelissen *et al*., 2010). Transcriptome analysis in those organisms revealed that only a small set of genes is affected by Elongator depletion with little apparent overlap of functional categories (Krogan and Greenblatt, 2001; Close *et al*., 2006; Nelissen *et al*., 2010). This argues against a general role of Elongator in transcription elongation.

In *A. thaliana* several auxin-related genes were found to be differentially expressed in wild-type and *elo* mutants, and at least two of these genes, *SHY2/IAA3* and *LAX2*, were also affected in histone H3K14 acetylation, which is a predominant target of the ScELP3-HAT (Nelissen *et al*., 2010). The data were taken as evidence for a role of Elongator in transcription elongation of specific genes, which may explain the observed influences on cell proliferation and development in *elo* mutants. How specificity for these genes might be achieved is currently unknown. Because auxin signalling is specific to plants whereas neuron development is specific to animals studies on the role of Elongator in these diverse processes might reveal how a protein complex with (a) conserved molecular function(s) has evolved to fulfil these kingdom specific functions. We believe that the answer to these questions can be found at the cellular level.

Taken together, our data indicate that the Elongator complexes are structurally and functionally highly conserved between yeast and plants, such that yeast subunits can be replaced by their plant orthologues. This offers the opportunity to take advantage of the yeast model system in structure–function studies of Elongator complexes from other kingdoms. It is likely that eukaryotic Elongator complexes have a conserved biochemical activity. Based on reconstitution of U34 tRNA modification in yeast by plant Elongator subunits and the requirement of AtElp3/ELO3 for this modification in plants, we favour the view that this conserved activity is directly related to tRNA modification.

## Experimental procedures

### Yeast strains, media and general methods

Yeast strains used are listed in Table 1. Yeast was grown in rich media containing yeast extract, peptone and 2% dextrose (YPD) or 2% galactose (YPG) or on synthetic complete medium (SC) (Sherman, 1991).

**Table 1:** Yeast strains.

Strain	Description	Source/reference
*K. lactis*		
AWJ137	α*leu2 trp1*[k1^+^ k2^+^] killer and zymocin producer	Karin D. Breunig
*S. cerevisiae*		
FY1679-08A	*MAT*a *ura3-52 leu2Δ1 trp1-Δ63 his3-Δ200* GAL	Euroscarf
CMY307	as FY1679-08A, but *elp3Δ::natNT2*	This study
FFY3t	as FY1679-08A, but *ELP3-(cmyc)_3_::SpHIS3*	Frohloff *et al.* (2001)
CMY306	as FY1679-08A, but *elp1Δ::TRP1, elp3Δ::natNT2*	This study
FFY2/3-dt	as FY1679-08A, but *ELP2-(cmyc)_3_::SpHIS3, ELP3-(HA)_6_::KlTRP1*	Fichtner *et al.* (2002a)
FFY2/3-dt-1d	as FFY2/3-dt, but *elp1Δ::KlURA3*	Fichtner *et al.* (2002b)
FFY2/4dt	as FY1679-08A, but *ELP2-(cmyc)_3_::SpHIS3, KTI12-(HA)_6_::KlTRP1*	Fichtner *et al.* (2002a)
CMY300	as FFY2/4dt, but *elp1Δ::hphNT1*	This study
CMY301	as FFY2/4dt, but *elp3Δ::natNT2*	This study
CMY302	As FFY2/4dt, but *elp1Δ::hphNT1, elp3Δ::natNT2*	This study
FFY2/5dt	as FY1679-08A, but *ELP2-(cmyc)_3_::SpHIS3, ELP5-(HA)_6_::KlTRP1*	Frohloff *et al.* (2003)
CMY304	as FFY2/5dt, but *elp3Δ::natNT2*	This study
CMY305	as FFY2/5dt, but *elp1Δ::hphNT1, elp3Δ::natNT2*	This study
W303-1a	*MAT*a *ade2-1 his3-11, 15 leu2-3, -112 trp1-1 ura3-1 can1-100*	Laboratory stock
CMY135	as W303, but *elp3Δ::natNT2*	This study
CMY134	as W303, but *elp1Δ::hphNT1, elp3Δ::natNT2*	This study
UMY2893	*MATα SUP4 leu2-3,112 trp1-1 can1-100 ura3-1 ade2-1 his3-11,15*	Huang *et al.* (2005)
UMY2916	*MATα SUP4 leu2-3,112 trp1-1 can1-100 ura3-1 ade2-1 his3-11,15 elp3Δ::KanMX4*	Huang *et al.* (2005)
CMY160	as UMY2916, but *elp1Δ::HIS3*	This study

Thermosensitivity was assayed on YPD medium at 30 or 38°C for 2–3 days. Drug sensitivity was assayed at 30°C with 5 mM caffeine. *ade2-1* ochre stop codon suppression by the *SUP4* gene was tested as described (Huang *et al*., 2005). Yeast was transformed with plasmid DNA or polymerase chain reaction (PCR) products according to a previous protocol (Schiestl and Gietz, 1989). Primers are listed in Table S1.

### *Arabidopsis thaliana* strains and growth conditions

An *A. thaliana* homozygous T-DNA insertion mutant in At5g50230 (*AtELP1*) derived from GABI-Kat Line 636H02 and the corresponding wild type (Columbia ecotype) were analysed for tRNA modification. Total RNA was prepared from leaves of several independent plants grown under short day conditions.

### Plasmid constructions

pFF9, a YEplac195-based plasmid carrying *ELP3* has been described (Frohloff *et al*., 2001). *A. thaliana AtELP3/ELO3* was amplified from cDNA clone RAFL0811J12 (http://www.brc.riken.jp) with primers cDNAfw and cDNA2re inserting flanking *Sal*I restriction sites. The *Sal*I fragment was fused to the yeast *ADH1* promoter and integrated into YEplac195 to give YEpA4. The *AtELP3* open reading frame was verified by DNA sequencing. In yatELP3 the *ADH1* promoter and 5′ end of the *AtELP3* gene from YEpA4 was replaced after cleavage with *Hin*dIII and *Ag*eI by the yeast *ELP3* promoter and 5′ end using primers E3′-fw and E3′-re and pFF9 as template. yatELP3M is a triple c-myc-tagged variant of yatELP3. pFF13 is a YEplac181-based plasmid, carrying the yeast *ELP1* gene (Fichtner *et al*., 2002a). pDJ98 is a multicopy plasmid for galactose-regulated expression of the *AtELP1* gene carrying the selection markers *URA3* and *leu2*d (Chen *et al*., 2006). pJET13, carrying *FLAG-AtELP1* under control of the *GAL1* promoter was obtained by PCR amplification of the coding sequence from pFLAG-ELO2 (Z. Gong, unpublished) and cloned into *Sal*I-cleaved pCM22. In the latter, the *GAL1* promoter was introduced on a *Eco*RI-*Bam*HI fragment from plasmid pRB1438 (a kind gift from Mike Stark, University of Dundee, UK) into YEplac181 (*LEU2*). pCM22 served also as destination vector to clone untagged *AtELP1* from pDJ98 via *Sal*I restriction sites to obtain pCM26 for the *SUP4* suppression assay. The centromeric plasmid p424TDH (Mumberg *et al*., 1995) served as the destination vector for ELP3 variants in double complementation studies. p424TDH-ScElp3myc encodes a C-terminal triple c-myc-tagged variant of *ScELP3* (kindly provided by O. Onuma, Univ. Halle), p424TDH-AtElp3myc a C-terminal triple c-myc-tagged variant of *AtELP3.* PCR-mediated domain swap of the AtELP3 HAT domain (amino acids 436–565) into p424TDH-ScElp3myc (1–426) and of ScElp3-HAT (382–557) into p424TDH-AtElp3myc (1–390) resulted in plasmids pELP3-AtHAT and pAtELP3-ScHAT respectively.

### Yeast genetic manipulations

Defined *elp1*Δ and *elp3*Δ null alleles and genetic variants encoding hemagglutinin (HA_6_) or c-myc_3_ epitope-tagged proteins were obtained after transformation of PCR fragments generated with template plasmids containing the marker genes YDp-KlU (*URA3*), YDp-SpH (*HIS3*), pFA6a-hphNT1, pFA6a-natNT2 (for deletions) or pYM3 and pYM5 (for HA and c-myc epitope-tagging) (Knop *et al*., 1999; Frohloff *et al*., 2001; Jablonowski *et al*., 2001; Janke *et al*., 2004). Manipulations were verified by PCR and by killer eclipse assays (Kishida *et al*., 1996) to test for biological functionality.

### Elongator complementation studies in yeast

To analyse the function of *Arabidopsis ELO2* and *ELO3* encoded gene products, the γ-toxin sensitivity was assayed (Frohloff *et al*., 2001). In detail, strains CMY135 (*elp3*Δ) and CMY134 (*elp1*Δ*elp3*Δ) were transformed with pDJ98 (*ELO2*/*AtELP1*), YEpA4 (*ELO3/AtELP3*), pFF13 (*ELP1*) and pFF9 (*ELP3*) (Frohloff *et al*., 2001; Fichtner *et al*., 2002a; Chen *et al*., 2006) or the respective empty vector controls. Subsequently, plasmid pHMS14 (Frohloff *et al*., 2001) expressing the γ-toxin subunit of *K. lactis* zymocin under the *GAL1* promoter was introduced into the transformed strains. Strains were grown on 2% (w/v) glucose SC medium under selective conditions and 10-fold serial dilutions were spotted on glucose and galactose medium. The response to γ-toxin induction was monitored on galactose plates after 3 to 4 days at 30°C.

### Immunological techniques

Detection of tagged proteins used anti-c-myc (A-14) and anti-HA (F-7) (Santa Cruz) antibodies. Anti-Elp1 antibodies (Otero *et al*., 1999; Wittschieben *et al*., 1999) were kindly provided by Dr J. Svejstrup (London Research Institute, Cancer Research, UK). An anti-FLAG antibody (Sigma–Aldrich) was used for detection of FLAG-ELO2. Protein concentrations were determined by the method of Bradford (Bradford, 1976). Antibody cross-linking to protein A-Sepharose, preparation of protein extract and co-immunoprecipitations were performed as described previously (Zachariae *et al*., 1996; Frohloff *et al*., 2001).

### tRNA isolation and HPLC analysis

tRNA was prepared from total RNA preparations as described (Bjork *et al*., 2001). Purified tRNA was digested with Nuclease P1 for 16 h at 37°C and then treated with bacterial alkaline phosphatase for 2 h at 37°C. The hydrolysate was analysed by high-pressure liquid chromatography with a Develosil C-30 reverse-phase column as described (Gehrke *et al*., 1982; Gehrke and Kuo, 1990).
