# Supplementary material for: Elongator function in tRNA wobble uridine modification is conserved between yeast and plants
Source: Mol Microbiol. 2010 Apr 26;76(5):1082–94. doi: 10.1111/j.1365-2958.2010.07163.x (PMC2904499; doi:10.1111/j.1365-2958.2010.07163.x)
Supplement: Supplementary file 1 [file mmi0076-1082-SD1.pdf]

## Supplemental Table S1. Oligonucleotide primers used in this study

| Name | Description | Sequence |
|---|---|---|
| S1-ELP1 | ko-primer | 5’-CGATGCCTAGGCAAGAAACAGTACAAATGCCTAATGGCTTATGGTTGAACCGTACGCTGCAGGTCGAC-3’ |
| S2-ELP1 | ko-primer | 5’-CTTTACGAGCACTATAGACAGTAATTTATATAACTAAGAAAATGGTATGCATCGATGAATTCGAGCTCG-3’ |
| S1-ELP3 | ko-primer | 5’-CCTAAAAGCACCTAAGGAAAATCGAAGAACACCCTGACAAAGATGGCTCGCGTACGCTGCAGGTCGAC-3’ |
| S2-ELP3 | ko-primer | 5’-CTGCTTGGAAAACCGGCCATGTCGGCGGCACATAAAAGTTCTATTTACCTATCGATGAATTCGAGCTCG-3’ |
| KO-ELP1-FW | ko-primer | 5’-AGAAACAGTACAAATGCCTAATGGCTTATGGTTGAACATGACAAGAGTGGCGACGGCCAGTGAATTCCCGG-3’ |
| KO-ELP1-RV | ko-primer | 5’-CAATATGACTCTTAGGGAAATCATGAATCTCTGGAACAGGTATTTCTGGGAGCTTGGCTGCAGGTCGACGG-3’ |
| ORF-ELP1-FW | ORF-primer | 5‘-ACCCGGGGATGGTTGAACATGACAAGAGTG-3‘ |
| ORF-ELP1-RV | ORF-primer | 5’-CAGATCTTTTGAATTCTGTTTCACGGCAGC-3’ |
| ORF-ELP3-FW | ORF-primer | 5’-CGATAAGACAGTGAGAGAAGG-3’ |
| ORF-ELP3-RV | ORF-primer | 5’-AACACATGCAGCAGTTACTCC-3’ |
| XFLAG-FW | *FLAG-ELO2* cloning | 5’-GCTCGAGCACCAAATCGACTCTAGGATGGC-3’ |
| XABO-RV | *FLAG-ELO2* cloning | 5’-GCTCGAGAACGATCGGGGGAGCTCTCATGG-3’ |
| cDNAfw | *ADH1-AtELP3* cloning | 5’- TATGTCGACTAGTATGGCGACGGCGGT-3’ |
| cDNA2re | *ADH1-ELP3* cloning | 5’-ATAGTCGACTCAAAGATGCTTCACC -3’ |
| HAT_FW | *ScElp3-AtHAT* | 5’-ATCCAAGAAGTGCATCATAAAGTTCAACCAGAACAAGTAGAGCTTGTGCG-3’ |
| HAT_RW | *ScElp3-AtHAT* | 5’-TCTAGAGGATCCGTCGACCTGCAGCGTACGAAGAAGATGCTTCACCATGT-3’ |
| SAM_FW | *AtElp3-ScHAT* | 5’-ATCAAGCTTATCGATACCGTCGACAAGCTTATGGCGACGGCGGTAGTGAT-3’ |
| SAM_BstEII_RV | *AtElp3-ScHAT* | 5’-GTTACCATTGTCAACACCTGAGGTAACCAAAGGCATAGGAATATCACGCT-3’ |
| atMyc-fw | *AtELO3-*$myc_3$ | 5’-AACAGCTGTACCAGTTCATGG -3’ |
| atMyc-re | *AtELO3-*$myc_3$ | 5’- TGAAGGCGCCCTAGACTCTAGA-3’ |
| E3’-fw | *ScELP3*-promoter | 5’-TACGCCAAGCTTGGTACC-3’ |
| E3’-re | *ScELP3*-promoter | 5’-ATAACCGGTTTAGCCTTCAATTTG-3’ |
